# Chewing Efficiency Test in Subjects with Clear Aligners

**DOI:** 10.3390/dj11030068

**Published:** 2023-03-01

**Authors:** Luca Levrini, Salvatore Bocchieri, Federico Mauceri, Stefano Saran, Andrea Carganico, Piero Antonio Zecca, Marzia Segù

**Affiliations:** 1 Department of Human Sciences and Innovation for the Territory, University of Insubria, 21100 Varese, Italy; 2 Department of Medicine and Surgery, University of Insubria, 21100 Varese, Italy; 3 Independent Researcher, 10121 Torino, Italy; 4 Department of Medicine and Surgery, University of Parma, 43126 Parma, Italy

**Keywords:** mastication, masticatory efficiency, oral function, clear aligners

## Abstract

The aim of this study was to evaluate the masticatory function of subjects with clear aligners and to propose a simple and repeatable method for the clinical and experimental evaluation of masticatory function. For the testing we used almonds, a natural substance that can be easily found and stored, has intermediate consistency and hardness, is insoluble in saliva, and has the ability easily lose the moisture absorbed in the mouth. Thirty-four subjects using the Invisalign^®^ (Align Technology, Santa Clara, CA, USA) protocol were randomly selected. This was an “intercontrol test”, i.e., all subjects under the same conditions acted as controls but also as cases whilst wearing the clear aligners. Patients were asked to chew an almond for 20 s, once with aligners and once without aligners. The material was then dried, sieved, and weighted. Statistical analysis was performed to investigate any significative differences. In all our subjects, the efficiency of chewing with clear aligners was found to be comparable to the efficiency of chewing without clear aligners. In detail, the average weight after drying was 0.62 g without aligners and 0.69 g with aligners, while after sieving at 1 mm, the average weight was 0.08 g without aligners and 0.06 g with aligners. The average variation after drying was of 12%, and after sieving at 1 mm, it was 25%. In summary, there was no substantial difference between chewing with or without clear aligners. Despite some discomfort in chewing, the clear aligners were well tolerated by most subjects, who wore them without difficulty even during meals.

## 1. Introduction

In orthodontics, functionality and aesthetics are the key factors in treatment with clear aligners. To ensure patient satisfaction, the orthodontist has to ensure that functional issues are solved while simultaneously paying attention to aesthetics during treatment [1]. Clear aligners have been increasingly accepted by patients due to their wearing comfort and aesthetics, as well as the fact that they are less noticeable and easier to clean than fixed braces [2]. The increased demand for orthodontic treatments among adults is probably related to the high availability of clear aligners that are less visible than traditional brackets [3].

Clear aligners, unlike other aesthetic orthodontic techniques, are removable, which aids the frequent cleaning of the devices, home hygiene measures, and eating. According to the manufacturers, they should be worn about 20–22 h a day. Orthodontic treatment, to be as effective as possible, should be continuous and constant over 24 h. Ideally, in order to achieve the highest efficiency, orthodontic pressure should be delivered constantly. However, clear aligners tend to be frequently removed from the mouth for meal consumption and oral hygiene practices, causing repeated interruptions in the efficiency of the devices. The manufacturer of the aligners used in this study suggests removing them only to eat and to brush and wear them for the rest of the day, because they are not supposed to create discomfort during daily life [3,4].

The evaluation of masticatory function should be carried out through standardized tests for every patient that receives dental treatment, especially when the occlusion is modified. It is also a factor that allows clinicians to check the success of a treatment [3,4,5]. Many oro-facial structures, such as the masticatory muscles, nerves, salivary glands, and teeth, are influenced by masticatory function [6]. Chewing or masticatory function also provides important support in reducing stress and cognitive functions, including alertness and executive functions. It is well known that chewing gum is used in the prevention of drowsiness during work, learning, and driving.

The assessment of masticatory function may involve subjective evaluation by the patient (masticatory ability) or objective test procedures (masticatory efficiency).

Surveys and interviews are useful to estimate subjective ability, but in order to obtain a quantifiable value, an objective test procedure must be used [7].

Various tests have been described in the literature that allow clinicians to evaluate the efficiency of the masticatory system. These tests can be performed using sieving techniques or by optical scanning, and both these approaches are considered adequate [8].

Regarding the sieving test, the use of multiple sieves evaluates the particle size obtained with high sensitivity: the smaller the size of the particles, the better the masticatory performance. In the past, a single sieve technique was adopted, but the multiple sieves approach is considered more detailed and reliable.

One of the most challenging aspects in the use of masticatory tests is to find and use appropriate specimens, i.e., choosing between mechanically degradable test foods or fragmenting tests that involve silicon cubes or wax and laboratory equipment [9].

Every solution has advantages and disadvantages; our proposal is a food test using almonds, since it is an easily stored and available material, sufficiently soft to be crushed even by patients with reduced chewing capacity, insoluble in saliva, and easily loses the moisture absorbed in the mouth during the test. Furthermore, the loss of material during the tests is modest and easily measurable.

The aim of this study was to evaluate the masticatory function of subjects with clear aligners and to propose a simple and repeatable method for the clinical and experimental evaluation of masticatory function. A test, therefore, was developed to evaluate the chewing effectiveness of patients.

## 2. Materials and Methods

This study was performed in accordance with the ethical standards established in the Declaration of Helsinki of 1975 and the Code of Ethics of the World Medical Association for experiments involving humans. Approval from the Ethics Committee (University of Insubria, protocol n°0111335 of 23 December 2022) was obtained. The informed consent of the participant was obtained prior to experimentation and the privacy rights of human subjects was always observed. A sample of 34 subjects was required in order to detect an effect size of 0.05 (statistical power set at 0,8). The sample of this study consisted of 34 subjects being treated with Invisalign^®^ (Align Technology, Tempe, AZ, USA). Patients were selected randomly among those in active treatment, with the following inclusion and exclusion criteria. Inclusion criteria: subjects aged 12 to 60 years, in complete permanent dentition (except for third molars, which may or may not have been present), treated with transparent aligners on both arches, and with a physiological chewing function. Exclusion criteria: subjects with an almond allergy, TMJ diseases, muscular issues that may affect chewing capacity, systemic diseases, and dental or periodontal decay.

**Figure 1:**
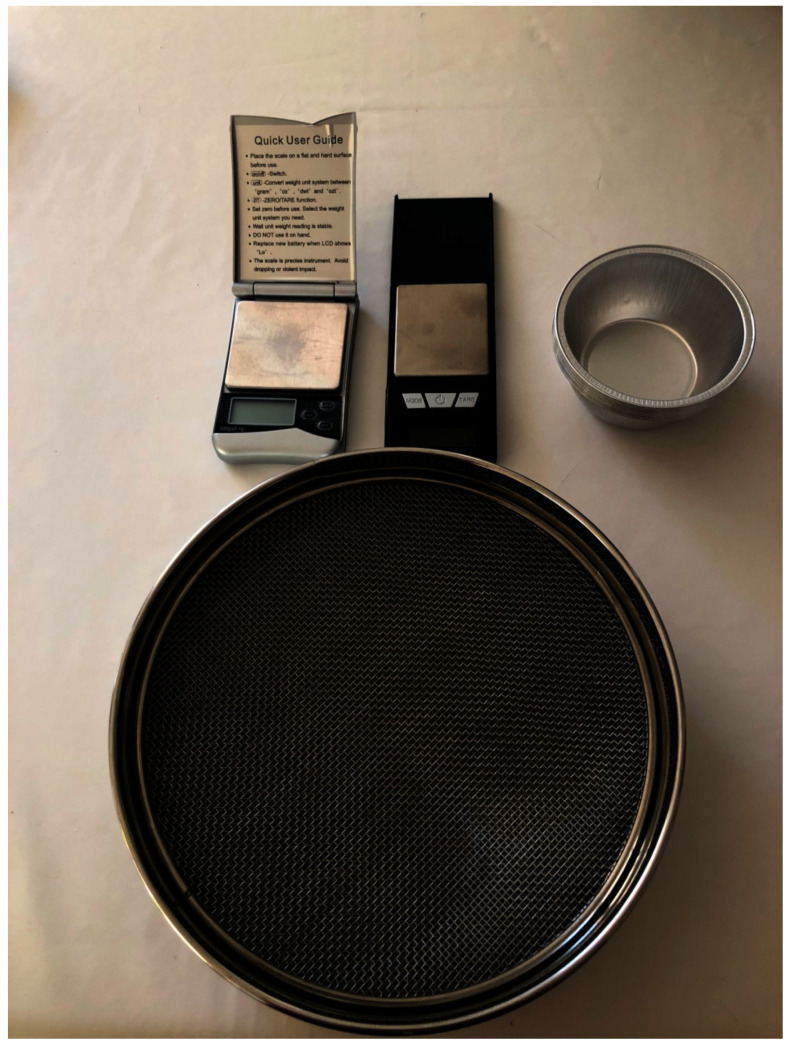
Precision scale and sieve used to collect and weight the specimens.

The subjects were asked to introduce a single almond (previously weighed) into the oral cavity and to chew it for 20 s. A trained operator calculated the time and counted the chewing cycles. The procedure was repeated with and without the aligners, allowing 5 min to pass between one measurement and the next. The patient was then asked to spit into a funnel with a fabric filter at the bottom. Before proceeding with the following step, the subject rinsed several times, and the oral cavity was carefully inspected by the operator to verify that no residues were left (Figure 1).

The material was then dried in the oven at 130° for 30 min to allow the almond to regain its initial hygrometry. A precision balance (Kenex^®^ 300 g/0.01 g) with a sensitivity of 0.01 g was used to weigh the residue in order to evaluate any loss of material due to the patient’s ingestion or any leak during the analysis procedure. Subsequently, the remaining particles were sieved through filters with meshes of decreasing size (4, 3, 2, and 1 mm), and the filtrates were weighed. The Shapiro–Wilk test was used to assess the normal distribution of the variables. A paired *t*-test was run to report any significant changes between the patients chewing with and without clear aligners (statistical significance was set at *p* < 0.05). All measurements were calculated as average and standard deviations.

## 3. Results

**Figure 2:**
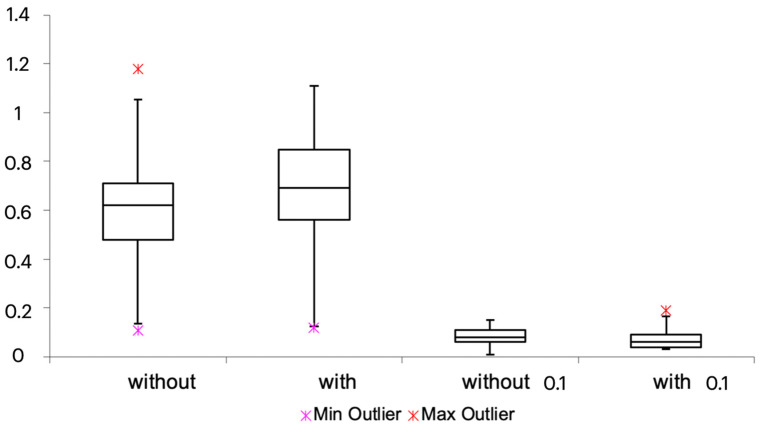
Graph of the average of the weights obtained.

For this study, 34 subjects, 17 males and 17 females, were enrolled. The average age was 25.6 years, and the average initial weight of the almond was 1.2 g. After the chewing cycle, the average weight of the residue was 0.69 g while wearing the clear aligner and 0.62 g without the aligner. After sieving at 1 mm, the average weight of the residues was 0.08 without aligners and 0.06 with aligners. An average loss of 12% was found after sieving compared to the weight after the drying process. After the filtering, the average weights of the dehydrated residues were 0.08 g and 0.06 g, respectively. After the sieving process, an average loss of 25% was recorded, as shown in Table 1 and Figure 2.

The statistical analysis showed no statistically significant difference between the two groups (*p*-values > 0.05), as shown in Table 2.

## 4. Discussion

Signals from numerous sensorial receptors in the orofacial structures determine the functional movements of the stomatognathic system and their respective forces. Periodontal mechanoreceptors, which have their sensory innervation in the periodontal ligament and are best positioned to detect the forces acting on the teeth, have a unique purpose. They are involved in mechanotransduction and chewing motor control, but there are still important limitations of knowledge in this field.

The mechanoreceptors are directionally sensitive. When a force is delivered to a tooth in one direction in humans, the periodontal mechanoreceptors respond with four to six separate afferents, but the direction of the stimulus sees the most activation. However, multiple tooth receptive fields do not affect the central nervous system’s ability to precisely pinpoint a particular tooth’s force stimulation. Additionally, periodontal afferents appear to have a propensity to saturate. The force rate required to split a peanut varies depending on the type of teeth involved, increasing distally along the dental arch with an average of 0.60 N for the incisor, 0.77 N for the canine, 1.15 N for the second premolar, and 1.74 N for the first molar. Regarding the threshold of perception of the force able to activate the receptor, the mean level detected in humans was below 1 N. Additionally, for all types of teeth, periodontal anesthesia increases the size and variety of the hold forces by at least a factor of two [10].

The chewing function has been investigated with various methods and in various categories of subjects. Electromyographic tests, spectrophotometers, optical scanners, and food tests were used to study masticatory efficiency in subjects with total prostheses, implant prostheses, or natural teeth in order to develop effective tests. Many methods, however, require expensive tools, high technical skills, and long procedures.

Regarding the materials developed specifically for tests, in 1980, Edlund et al. [2] used a silicone material for taking dental impressions (Optosil^®^, Bayer). The test led to an index of chewing efficiency and consisted of chewing a round tablet of Optosil^®^, 5 mm thick and 20 mm in diameter, for 20 chewing cycles, after which the subject had to spit the chewed part into a plastic container and rinse and empty their mouth into the same container. The contents were then filtered through sieves with 2.8, 1.9, and 1 mm diameter meshes and weighed. The efficiency was considered high if the weight of the material passing through the 1 mm filter was higher in percentage compared to the weight of the material which had passed through the other two filters.

In 1983, Gunne [11] developed a new method by dissolving 60 mg of erythrosine in 20 mL of water and adding it to a solution of 1180 mL of gelatin, which was then hardened in 2.3% formalin for 24 h. Finally, using a mold, the gelatin was divided into about 54 cubes, weighing 9.39 g in total. The cubes were then chewed for as long as the patient wished, and then was collected in a plastic container that had a 0.45 mm mesh filter at the bottom, and the chewed part was then rinsed with running water to remove any residual saliva. Finally, the chewed part was placed in a water-soluble pigment, which penetrated an average of 0.15 mm into the gelatin and resulted in a decrease in the concentration of the pigment in the container. This value was used to calculate chewing efficiency based on the fact that the more the pigment was spread in the jelly, the greater the area of the jelly exposed, and thus, the greater the chewing efficiency.

In 1989, Nakasima et al. [12] developed a test that involved the use of granules of 1 mm in diameter formed by erythrosine, cellulose, lactose, corn starch, sucrose, hydrogenated oil, and other materials, which were collected in quantities of 730 mg (approximately 730–740 granules) in a cylindrical capsule, 20 mm in length and 10 in diameter. The capsule was then chewed. The resulting particles were rinsed with distilled water, and the amount of erythrosine dissolved in the water was measured with a spectrophotometer.

Shi et al. [13] used soy, which was prepared for testing by soaking for 24 h in water, followed by a 5 min boil. Ten subjects with full natural dentition were selected and asked to chew 3 gm of soy for 10, 20, and 30 chewing cycles. The chewed part was analyzed with a microscope in order to photograph the chewed particles, the data obtained were entered in a special program (written by the same authors who developed the test) to calculate the diameter of the particles and the statistical values in order to determine whether the results obtained were statistically acceptable, and, therefore, whether the test was valid.

In 1994, Mowlana et al. [8] compared two methods: one based on a system of sieves, and one based on the use of digital scanners. Both methods were tested on six subjects between 21 and 29 years of age with complete natural dentition, and almonds were used for the tests. The tests consisted of chewing an almond 4, 8, 16, and 32 times. The chewed almonds were then passed through a sieve system with a mesh diameter of between 0.5 and 8 mm and weighed, and the weight obtained was converted into volume since the specific mass of the material is known. In the other method, the chewed almonds were scanned in order to find the number and volume of the particles. The results showed that these methods are equally valid as a chewing test.

In 2007, Schimmel et al. [14] developed a test that used a two-colored chewing gum (blue and pink) 30 mm long, 18 mm wide, and 3 mm thick for 20 subjects aged 22 to 35 to chew. The chewing-gum was chewed for 20 chewing cycles, and then analyzed: the quantity of pure color not mixed was calculated using a graphic processing program that counted the pixels relating to the two basic colors of the chewing-gum. The results were statistically processed to understand whether this type of test was valid.

In 2010, Nokubi et al. [15] based a test on the use of gelatin in which β-carotene was dissolved, subsequently measured with light-emitting diodes and photodiodes that analyze the concentration of β-carotene that dissolves in water after chewing.

In 2012, Ikebe et al. [16] also developed a test that uses jellies containing glucose, which was released during chewing, and which was then measured by a glucometer in the aqueous solution in which the jellies were collected after the test. In addition, this particular test also collected other data, such as the salivary flow of patients, the maximum occlusal force, and the number of occlusal contacts.

A 2014 study by Oliveira et al. reviewed all the methodologies for assessing masticatory efficiency [17]. The most used materials were found to be almonds for natural foods, and optical for artificial foods. A sieve system for measuring was found to be the easiest in terms of setting up and availability, as well as its provision of very precise results.

In 2018, Shala et al. [18] evaluated chewing efficiency through the use of electromyography on a total of 88 patients with total mobile prostheses.

In 2019, in a pediatric study that aimed to evaluate the chewing function of patients with anterior open bite, Correa et al. [19] used colorimetric capsules as an evaluation method. The sample consisted of 106 patients aged 7 to 11 years, 51 of whom had an anterior open bite (experimental group) and 55 had a normal bite (control group). The color capsules used in this evaluation contained fuchsin granules, which were used to create a solution after being ground during chewing. Each patient was asked to chew one capsule for 20 s. The solutions obtained were analyzed with a spectrophotometer. Children with an anterior open bite showed a lower chewing efficiency than those with a normal bite.

In the Glossary of Prosthodontic Terms, masticatory performance is described as “a measure of the comminution of food attainable under standardised testing settings”, while “masticatory efficiency” is described as “the effort necessary to obtain a standard degree of comminution of food”.

That being said, a proper definition of masticatory performance is still unclear and poorly supported by scientific evidence.

According to Bates et al., while chewing efficiency refers to the number of chewing cycles required to achieve a specific chewing outcome, chewing performance refers to the status of the chewing outcome after a specific number of chewing cycles. In other words, whereas masticatory efficiency refers to the number of chewing cycles required to achieve half the original particle size, masticatory performance refers to the ability of the individual to grind or pulverize a specimen of test food after a predetermined number of mastication cycles [20].

Although there is disagreement among scholars on the precise meaning of each phrase, related names are occasionally used to denote other approaches. This could result in comparisons between various test methodologies, endangering the validity of scientific data of physiological or therapeutic treatments [17].

So far, no author has studied the chewing function of patients with and without clear aligners.

In this study, a simple and repeatable clinical and experimental method was proposed for the evaluation of the chewing function and, at the same time, to assess the chewing function of subjects with clear aligners. The aim of the study was to investigate whether this type of device can also be worn during meals, which would then increase the time of use of these devices and, therefore, the effectiveness of the treatment.

The need for aesthetic orthodontic procedures, such as clear aligners, is rising today in both adults and adolescents. Numerous studies have underlined the higher aesthetic and comfort provided by clear aligners when compared to traditional fixed orthodontics. In recent years, phase one treatment is also starting to be performed with clear aligners.

Compared with traditional fixed orthodontic appliances, clear aligners are considered a more aesthetic and comfortable alternative [21]. In addition, the use of removable appliances reduces the negative effects of fixed orthodontics on the periodontal tissues and allow for better oral hygiene [22].

Comfort surely represents one of the most important aspects of clear aligners techniques: even though the need for removing aligners for meal consumption is a limit in this perspective, the comfort of chewing with aligners still has to be evaluated.

In line with the findings of Oliveira et al. [17], almonds were used for the current investigation. Almonds are readily available and easy to store. They can be crushed sufficiently even by patients with reduced chewing capacity, they are insoluble in saliva, and they easily lose the moisture acquired in the oral cavity during the test. Moreover, not much material is lost during the tests and it is easy to measure.

Although techniques using optical scanners can test many variables and conditions more quickly, a food test with the sieving of the chewed food was utilized in this study. This was because previous studies have shown that food tests entail using less expensive equipment and are easier to use in clinical practice [9], but at the same time give equally reliable results compared to more complex tests [6].

In this research, in all 34 subjects, the difference between the weighed values of the chewed almonds with and without aligners was slight, with no statistically significant differences between the two groups analyzed.

In our study, a sample of patients who presented a complete set of teeth was selected. As reported by Alves et al., the number of remaining teeth needs specific consideration, since tooth loss influences the sensory and motor components of the masticatory process. Mechanoreceptor loss has been documented along with tooth loss. Periodontal mechanoreceptors are neural receptors that help the masticatory muscles contract and work together. Therefore, the loss of sensory feedback and subsequent muscle atrophy following tooth loss reduced the ability to masticate [23].

In this study, almonds were chosen as the brittle food for the comminution test. Other options are raw carrots or nuts. When chewed, food breaks up into smaller pieces, creating the food bolus. Sieving or optical scanning can be used to analyze the food particles. The distribution of particle sizes in the chewed food sample is typically described in terms of the median particle size. Masticatory performance is the outcome of chewing food for a predetermined number of chewing cycles. After spitting and drying the bolus into a different sizes sieves, the remaining particles were counted and evaluated with a statistical analysis.

There are several reasons why a comminution test was chosen. The human masticatory system has evolved to allow the chewing of both tough foods, such as meat, and brittle solid foods, such as almonds and carrots. A person’s ability to chew a brittle solid food is correlated with their ability to chew a variety of foods that are either harder or less tough. Additionally, multiple research projects have effectively used comminution tests to quantify masticatory function. These tests make it possible to assess both chewing effectiveness and performance. Comminution tests are an accurate approach to measuring how well an individual or a group can chew. More in-depth data about the chewing process was obtained for the masticatory efficiency as a result of multiple tests carried out after varying the number of chewing cycles, providing a comparison of inter-subjects at the same stage of food comminution and constant intra-subject and inter-subject ratios between and within samples, respectively [24].

According to this study, the capability to chew food while wearing aligners is valid and shows encouraging results, which highlight the possibility of wearing aligners continuously throughout the day.

Various aspects still need to be investigated in a more detailed way, such as the quantification of the effective therapeutic advantage derived from the continuous use of the clear aligners in 24 h, and the properties of resistance and deformability of the material that make up the clear aligners themselves. If the clear aligners are worn during meals, they should be able to resist the sudden changes in temperature caused by the ingestion of food and should also be non-deformable under the subjected chewing forces.

Moreover, the implications that the aligner worn 24 h could have should be investigated, since it would further reduce the natural cleansing of the saliva on the tooth [25].

Some limitations of the study are that patients were not asked to report their level of annoyance while chewing with the clear aligners. Further studies should also evaluate whether the swallowing function and the masticatory pattern can be influenced by wearing aligners during meals. Additionally, the small sample size and the possible variability in almond size and weight could affect the conclusions of this study.

## 5. Conclusions

The results highlighted no substantial difference between chewing with and without clear aligners from a chewing point of view.

With regard to the food test used, this was found to be quick and simple to perform and equally fast to analyze and was tolerated even by the youngest subjects. The almond was also suitable for this type of experiment, considering its intermediate consistency and hardness, its availability, low cost, its ability easily loses the moisture absorbed in the mouth, and its good level of tolerance by the subjects.

Clear aligners could be worn during meals to maximize the therapeutic effect, since this could lead to an increase in aligner wearing time, but furthers studies are needed to investigate and confirm these hypotheses. Researchers should also investigate any variation in aligner fit or advantages and comfort during meals.

## Figures and Tables

**Table 1:** Descriptive statistics for data. “without” means weight after drying without clear aligner; “with” means weight after drying with clear aligner; “without 1 mm” means weight after sieving at 1 mm without clear aligner; “with 1 mm” means weight after sieving at 1 mm with clear aligner.

Labels	Without	With	Without 1 mm	With 1 mm
Min	0.11	0.12	0.01	0.03
Q1	0.48	0.56	0.06	0.04
Mean	0.62	0.69	0.08	0.06
Q3	0.71	0.85	0.11	0.09
Max	1.18	1.11	0.15	0.19
IQR	0.23	0.29	0.05	0.05
Upper outliers	1	0	0	1
Lower outliers	1	1	0	0

**Table 2:** Standard deviations and *t*-test of data. “without” means weight after drying without clear aligner; “with” means weight after drying with clear aligner; “without 1 mm” means weight after sieving at 1 mm without clear aligner; “with 1 mm” means weight after sieving at 1 mm with clear aligner.

Without	With	*p*-Value	Without 1 mm	With 1 mm	*p*-Value
0.62 ± 0.21	0.69 ± 0.21	*p* > 0.05	0.08 ± 0.03	0.06 ± 0.03	*p* > 0.05

## Data Availability

Data are available from the corresponding author upon reasonable request.

## References

[1] Alshammari A., Almotairy N., Kumar A., Grigoriadis A. (2022). Effect of malocclusion on jaw motor function and chewing in children: A systematic review. Clin. Oral Investig..

[2] Edlund J., Lamm C.J. (1980). Masticatory efficiency. J. Oral Rehabil..

[3] de Abreu R.A., Pereira M.D., Furtado F., Prado G.P., Mestriner W., Ferreira L.M. (2014). Masticatory efficiency and bite force in individuals with normal occlusion. Arch. Oral Biol..

[4] Shim J., Ho K.C.J., Shim B.C., Metaxas A., Somogyi-Ganss E., Di Sipio R., Cioffi I. (2020). Impact of post-orthodontic dental occlusion on masticatory performance and chewing efficiency. Eur. J. Orthod..

[5] Akeel R., Nilner M., Nilner K. (1992). Masticatory efficiency in individuals with natural dentition. Swed. Dent. J..

[6] Nakata M. (1998). Masticatory function and its effects on general health. Int. Dent. J..

[7] Boretti G., Bickel M., Geering A.H. (1995). A review of masticatory ability and efficiency. J. Prosthet. Dent..

[8] Mowlana F., Heath M.R., Van der Bilt A., Van der Glas H.W. (1994). Assessment of chewing efficiency: A comparison of particle size distribution determined using optical scanning and sieving of almonds. J. Oral Rehabil..

[9] Woda A., Nicolas E., Mishellany-Dutour A., Hennequin M., Mazille M.N., Veyrune J.L., Peyron M.A. (2010). The masticatory normative indicator. J. Dent. Res..

[10] Piancino M.G., Isola G., Cannavale R., Cutroneo G., Vermiglio G., Bracco P., Anastasi G.P. (2017). From periodontal mechanoreceptors to chewing motor control: A systematic review. Arch. Oral Biol..

[11] Gunne H.S. (1985). Masticatory efficiency and dental state. A comparison between two methods. Acta Odontol. Scand..

[12] Nakasima A., Higashi K., Ichinose M. (1989). A new, simple and accurate method for evaluating masticatory ability. J. Oral Rehabil..

[13] Shi C.S., Guan Q.Y., Guo T.W. (1990). Masticatory efficiency determined with direct measurement of food particles masticated by subjects with natural dentitions. J. Prosthet. Dent..

[14] Schimmel M., Christou P., Herrmann F., Muller F. (2007). A two-colour chewing gum test for masticatory efficiency: Development of different assessment methods. J. Oral Rehabil..

[15] Nokubi T., Nokubi F., Yoshimuta Y., Ikebe K., Ono T., Maeda Y. (2010). Measuring masticatory performance using a new device and beta-carotene in test gummy jelly. J. Oral Rehabil..

[16] Ikebe K., Matsuda K., Kagawa R., Enoki K., Okada T., Yoshida M., Maeda Y. (2012). Masticatory performance in older subjects with varying degrees of tooth loss. J. Dent..

[17] Oliveira N.M., Shaddox L.M., Toda C., Paleari A.G., Pero A.C., Compagnoni M.A. (2014). Methods for evaluation of masticatory efficiency in conventional complete denture wearers: A systematized review. Oral Health Dent. Manag..

[18] Shala K., Bicaj T., Pustina-Krasniqi T., Ahmedi E., Dula L., Lila-Krasniqi Z. (2018). Evaluation of the Masticatory Efficiency at the Patients with New Complete Dentures. Open Access Maced. J. Med. Sci..

[19] Correa E.C., Maeda F.A., de Miranda A.L.R., Carvalho P.E.G., da Silva L.H., Torres F.C. (2018). Masticatory evaluation of anterior open bite malocclusion using the colorimetric capsule method. Gen. Dent..

[20] Bates J.F., Stafford G.D., Harrison A. (1976). Masticatory function—A review of the literature. III. Masticatory performance and efficiency. J. Oral Rehabil..

[21] White D.W., Julien K.C., Jacob H., Campbell P.M., Buschang P.H. (2017). Discomfort associated with Invisalign and traditional brackets: A randomized, prospective trial. Angle Orthod..

[22] Jiang Q., Li J., Mei L., Du J., Levrini L., Abbate G.M., Li H. (2018). Periodontal health during orthodontic treatment with clear aligners and fixed appliances: A meta-analysis. J. Am. Dent. Assoc..

[23] Alves C.P., Munhoz M.F.V., Oliveira Nascimento G.M., Nicoli G.A., Paleari A.G., Camargos G.V. (2019). The Influence of Age, Gender, Mandibular Bone Height, Previous Experience with Prostheses, and Fabrication Methods on Masticatory Performance of Complete Denture Wearers. J. Prosthodont..

[24] Goncalves T., Schimmel M., van der Bilt A., Chen J., van der Glas H.W., Kohyama K., Hennequin M., Peyron M.A., Woda A., Leles C.R. (2021). Consensus on the terminologies and methodologies for masticatory assessment. J. Oral Rehabil..

[25] Levrini L., Novara F., Margherini S., Tenconi C., Raspanti M. (2015). Scanning electron microscopy analysis of the growth of dental plaque on the surfaces of removable orthodontic aligners after the use of different cleaning methods. Clin. Cosmet. Investig. Dent..

